# Immunoproteasome acted as immunotherapy ‘coffee companion’ in advanced carcinoma therapy

**DOI:** 10.3389/fimmu.2024.1464267

**Published:** 2024-08-30

**Authors:** Shaoyan Shi, Xuehai Ou, Chao Liu, Hao Wen, Ke Jiang

**Affiliations:** Department of Hand Surgery, Honghui Hospital, Xi’an Jiaotong University, Xi’an, Shaanxi, China

**Keywords:** immunoproteasome, immunotherapy, solid tumors, immune, immune checkpoint inhibitors

## Abstract

Immunoproteasome is a specialized form of proteasome which plays a crucial role in antigen processing and presentation, and enhances immune responses against malignant cells. This review explores the role of immunoproteasome in the anti-tumor immune responses, including immune surveillance and modulation of the tumor microenvironment, as well as its potential as a target for cancer immunotherapy. Furthermore, we have also discussed the therapeutic potential of immunoproteasome inhibitors, strategies to enhance antigen presentation and combination therapies. The ongoing trials and case studies in urology, melanoma, lung, colorectal, and breast cancers have also been summarized. Finally, the challenges facing clinical translation of immunoproteasome-targeted therapies, such as toxicity and resistance mechanisms, and the future research directions have been addressed. This review underscores the significance of targeting the immunoproteasome in combination with other immunotherapies for solid tumors and its potential broader applications in other diseases.

## 1 Introduction

**Figure 1:**
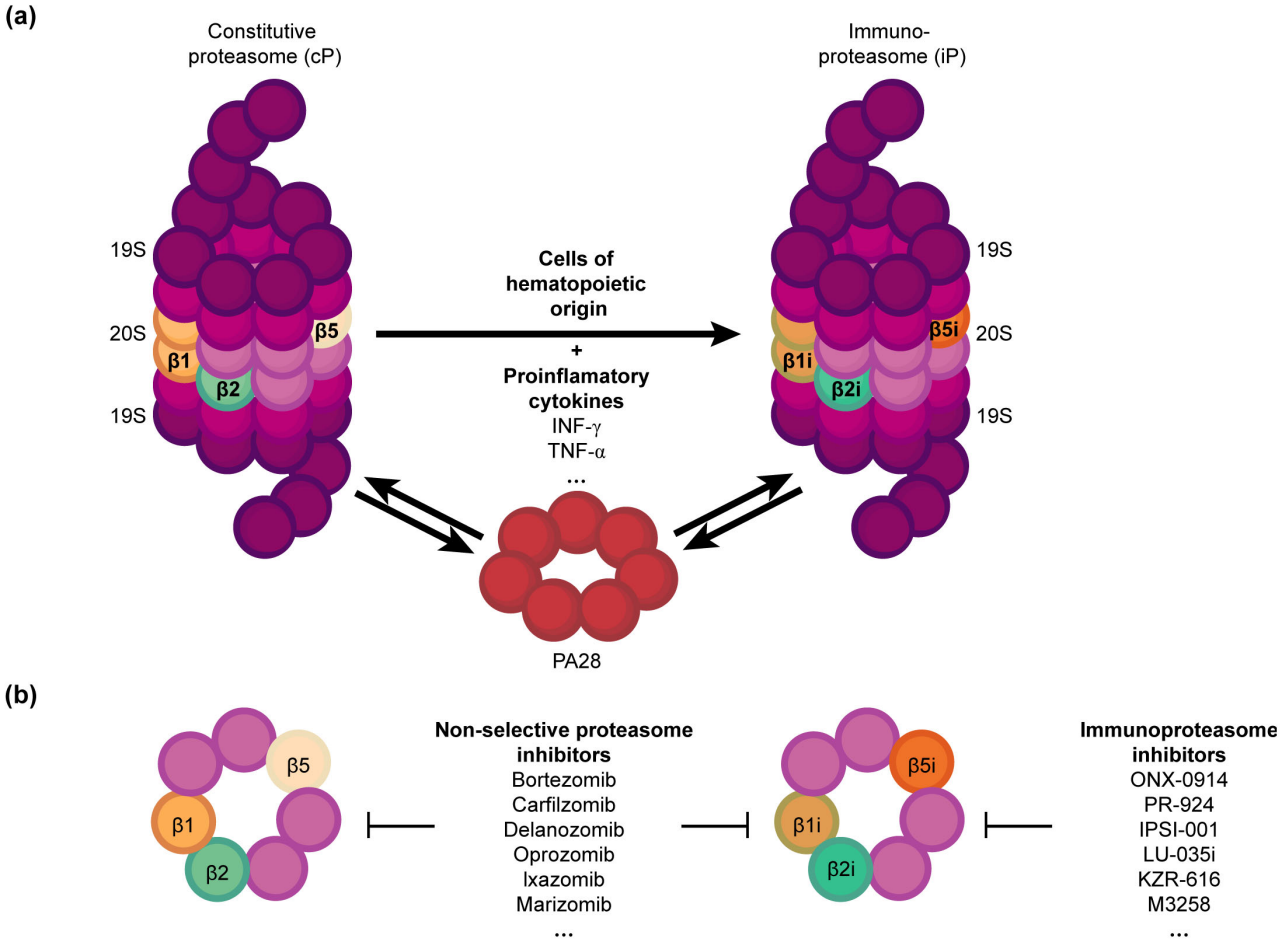
Structure and function of proteasomes **(A)** Constitutive proteasome (cP) vs. Immunoproteasome (iP): The cP includes the subunits β1, β2 and β5, which are respectively substituted with β1i, β2i, and β5i in the iP upon stimulation by pro-inflammatory cytokines such as IFN-γ. The iP is naturally present in hematopoietic cells. IFN-γ also upregulates the regulatory particle PA28, which can bind to both the cP and iP with equal affinity and enhance their activity. **(B)** Proteasome inhibitors can selectively target iP, or both cP and iP, offering potential immunomodulatory and therapeutic strategies against cancer.

The immunoproteasome is a variant of the standard proteasome with distinct catalytic subunits that degrade ubiquitinylated proteins (1, 2). It is activated in both immune and non-immune cells in response to inflammatory cytokines, especially interferon-gamma (IFN-γ), and oxidative stress (3, 4). Nevertheless, the primary function of the immunoproteasome is to cleave intracellular viral or oncogenic proteins into peptides, which are then displayed by the major histocompatibility complex (MHC) class I molecules for CD8+ T cells (1, 5). The immunoproteasome differs from the standard proteasome in terms of both enzymatic activity and subunit composition (6, 7). The β1, β2, and β5 catalytic subunits of the standard proteasome (8, 9) are respectively substituted with β1i (LMP2), β2i (MECL-1), and β5i (LMP7) in the immunoproteasome (1, 10) (**Figure 1**). These substitutions alter the proteolytic activity of the immunoproteasome, allowing efficient production of peptides with hydrophobic or basic C-terminal residues, which are preferentially bound by the MHC class I molecules for antigen presentation (2, 11, 12). Thus, immunoproteasomes boosts the ability of the immune system to recognize and respond to intracellular infections and tumor cells by increasing the efficacy and specificity of peptide generation.

Solid tumors are abnormal masses of tissue that can be benign (non-cancerous) or malignant (cancerous) (13, 14). Depending on the tissue of origin, solid tumors are broadly classified as carcinomas that originate from epithelial cells (e.g., breast, lung, colorectal cancers), and sarcomas that arise from connective tissues (e.g., bones, muscles). Other specific types include gliomas (brain) and hepatomas (liver) (15–17). Solid tumors are often detected in the advanced stages, which can render the conventional therapies, such as radiation therapy, chemotherapy and surgery, less effective (18–20). In addition, solid tumors frequently develop resistance to these therapies, leading to recurrence and metastasis. The presence of immune cells, blood vessels, and the extracellular matrix in the tumor microenvironment (TME) significantly influences tumor progression and treatment resistance. The heterogeneity of solid tumors further complicates treatment, as different regions of the same tumor can respond differently to the same therapeutic modality. These challenges underscore the need for novel approaches, including immunotherapy.

Immunotherapy harnesses the host immune system to recognize and eliminate cancer cells. For instance, checkpoint inhibitors can augment the anti-tumor immune response by reversing the inhibitory signals on the effector T cells. Likewise, immune cells engineered to effectively recognize and eliminate tumor cells (i.e., adoptive cell transfer) and cytokines have also been shown to enhance immune-based clearance of the malignant cells. Furthermore, cancer vaccines can stimulate the immune system to target specific tumor antigens (21–25). However, tumor cells have developed adaptive mechanisms to escape immune detection, such as decreasing antigen presentation or inducing an immunosuppressive TME. Immunotherapeutic strategies can overcome these challenges by promoting the recognition of tumor-specific antigens. In addition, immunotherapies also offer the potential for long-lasting protection against cancer by establishing immunological memory, as well as a viable alternative for tumors that are resistant to conventional therapy. The therapeutic potential of targeting immune-related pathways has been highlighted by the success of checkpoint inhibitors against melanoma and lung cancer among other malignancies (26–28).

In this review, we have explored the role of immunoproteasome in anti-tumor immune responses and its potential as a therapeutic target for cancer. We have also discussed the current research, clinical applications, and the challenges associated with targeting the immunoproteasome in solid tumors, and identified areas for future research and clinical development.

## 2 Structure and function of immunoproteasomes

The immunoproteasome differs from the standard proteasome on account of three inducible catalytic subunits. The β1i subunit, also referred to as low molecular weight protein 2 (LMP2), is encoded by a gene located within the MHC class II locus, which emphasizes its significant role in immune function (29, 30). Unlike the β1 subunit, LMP2 primarily generates peptides with hydrophobic C-termini, which are preferred by MHC class I molecules. LMP2 is upregulated by inflammatory cytokines, which ensures that the immunoproteasome is optimized for processing antigens during immune responses (31, 32). The β2 subunit of the standard proteasome is replaced with the β2i subunit, also referred to as multi-catalytic endopeptidase complex-like 1 (MECL-1) (10, 33). MECL-1 enhances cleavage of substrates after basic residues, which generates a broader range of antigenic peptides suitable for MHC class I presentation. The induction of MECL-1 by pro-inflammatory cytokines is an essential step for the activation of CD8+ T cells and the ensuing adaptive immune response (34, 35). Furthermore, the presence of MECL-1 is essential for maintaining the unique substrate specificity of immunoproteasomes. The β5i subunit, also known as LMP7, is the third inducible catalytic subunit of the immunoproteasome, and promotes the generation of hydrophobic peptides or those with basic C-terminal residues. The gene encoding LMP7 is also located in the MHC region, which underscores its role in immune function. It is induced by IFN-γ and other pro-inflammatory cytokines, and is necessary for the effective processing of tumor and viral antigens (36, 37).

IFN-γ is a key effector cytokine involved in the immune responses against viral infections and tumors. Exposure to IFN-γ leads to upregulation of immunoproteasome subunits in the immune cells (38). The tumor necrosis factor-alpha (TNF-α) also contributes to the immunoproteasome induction (39, 40). Both cytokines facilitate the assembly of the β1i, β2i, and β5i subunits into the immunoproteasome during immune responses. Various chaperones are involved in this process to ensure correct incorporation and folding of the subunits (41–43). This regulated assembly ensures that the immunoproteasome is formed efficiently during times of immune activation, providing a tailored response to pathogenic challenges (44–46).

The transporter associated with antigen processing (TAP) cleaves proteins into smaller peptides during protein degradation, which are then transported into the endoplasmic reticulum (47, 48). Within the endoplasmic reticulum, these peptides are loaded onto MHC class I molecules. When MHC class I molecules are present on the surface of malignant or infected cells, CD8+ T cells are able to recognize and respond to these antigens (49, 50).

## 3 Immunoproteasome in tumor immunology

### 3.1 Recognition and elimination of cancer cells

Immune cells have the ability to recognize and eliminate cells that express abnormal or cancer-specific antigens, a phenomenon known as immunosurveillance. The immunoproteasome is pivotal to this process, as it generates a wide repertoire of antigenic peptides that are more likely to be recognized by the effector immune cells. The presentation of these peptides by the MHC class I molecules is crucial for the activation of anti-tumor CD8+ cytotoxic T lymphocytes (CTLs) (11, 51). Consequently, the immunoproteasome plays a critical role in the prevention and control of cancer progression by assisting the immune system in detecting and eliminating nascent tumor cells.

Solid tumors have developed several mechanisms to avoid immune detection and clearance, even in the face of effective immune surveillance. For instance, tumor cells can downregulate the expression of MHC class I molecules, which reduces antigen presentation and subsequent recognition by CTLs. Additionally, tumors can create an immunosuppressive microenvironment by secreting factors such as TGF-β, IL-10, and VEGF (52). Likewise, immune checkpoint molecules like PD-L1 that cause T cell exhaustion by triggering inhibitory signals are also typically upregulated on the tumor cells. Finally, mutations or alterations in the immunoproteasome subunits can lower generation of antigenic peptides, and contribute to immune evasion.

### 3.2 Role of immunoproteasome in modulating the microenvironment

Apart from ensuring effective presentation of tumor-associated antigens (TAAs) on the MHC class I molecules and thus promoting activation of CTLs (53, 54), the immunoproteasome also maintains the balance between immune activation and tolerance, and keeps the immunosuppressive elements of the TME in check (35, 52). It can either promote or inhibit the anti-tumor immune response by modulating the activity of various immune cells and the production of cytokines.

Tumor-infiltrating lymphocytes (TILs) are a subset of immune cells that penetrate the tumor stroma, and are indicative of the immune response against malignant cells. Enhanced antigen processing by the immunoproteasome can boost the responsiveness and cytotoxic function of the TILs against tumor cells (55, 56), which is vital for sustained anti-tumor immune response within the TME. However, tumors often employ immunosuppressive strategies that can hinder TIL function, such as upregulation of checkpoint molecules and the secretion of immunosuppressive cytokines. Targeting the immunoproteasome within the TME can potentially counteract these strategies, and enhance the infiltration, activation, and cytotoxic function of TILs (57, 58).

### 3.3 Immunoproteasome activity in antigen-presenting cells

Antigen-presenting cells (APCs), including macrophages and dendritic cells (59, 60), process and present antigens to T cells, thereby initiating and modulating immune responses. The immunoproteasome enhances the production of high-quality antigenic peptides, which results in a wide and robust array of tumor-associated antigens being displayed on the surface of APCs. This, in turn, leads to a more effective activation and expansion of CTLs, promoting a stronger and more targeted antitumor immune response. Additionally, by affecting cytokine production and lowering immunosuppressive factors, the immunoproteasome can modify the tumor microenvironment and improve the immune response against tumors as a whole.

## 4 Immunoproteasome as a therapeutic target

### 4.1 Enhancing antigen presentation

**Figure 2:**
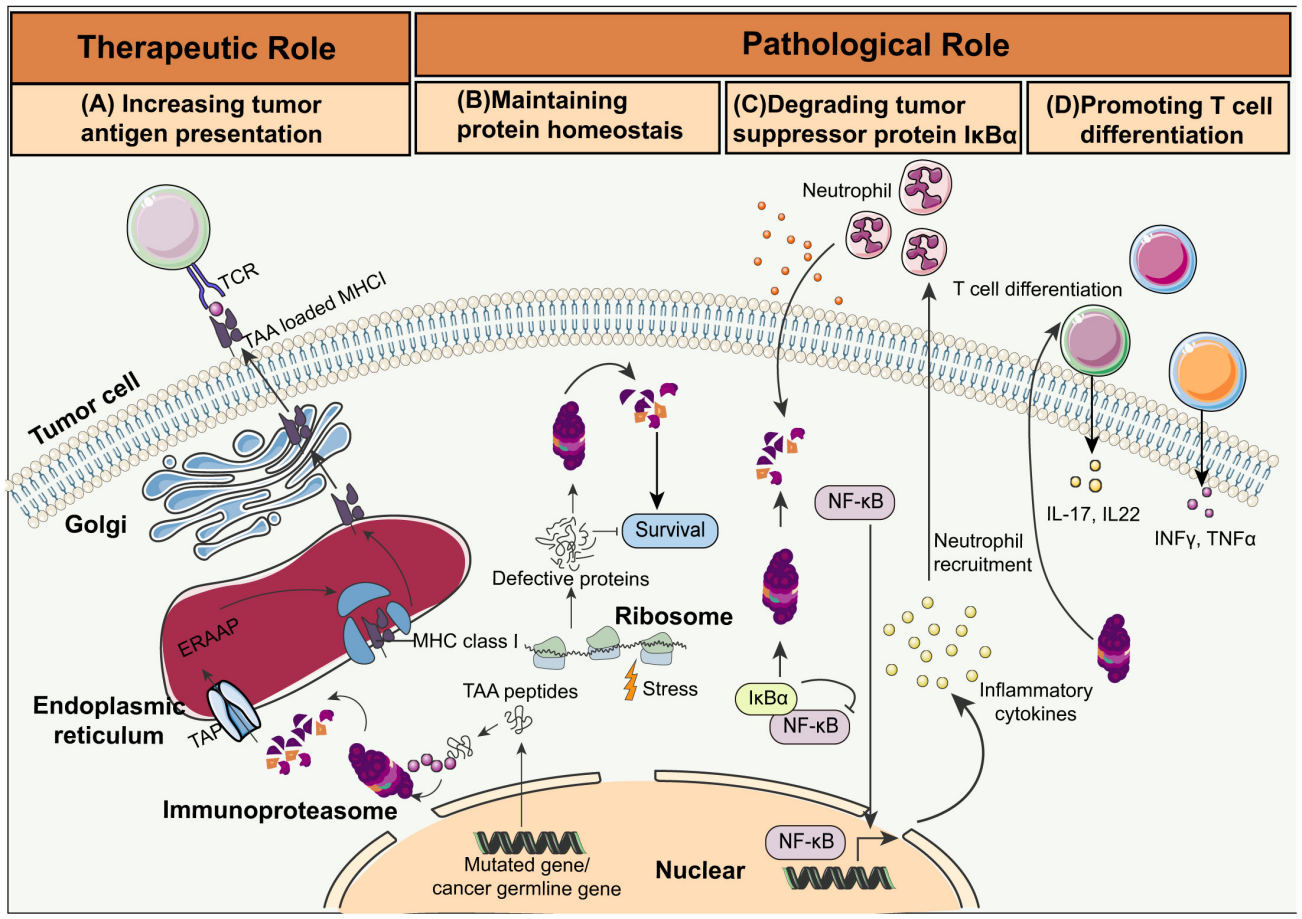
Schematic overview of immunoproteasome activities in cancer. **(A)** Increasing tumor antigen presentation: Immunoproteasomes degrade tumor-associated antigen (TAA) peptides encoded by mutated or cancer germline genes. The transporter associated with antigen processing (TAP) then delivers these peptides into the endoplasmic reticulum (ER). The TAA peptides are trimmed by the ER-associated aminopeptidase (ERAAP), and loaded on MHC I molecules by a complex of tapasin, calreticulin, and ERP57. The MHC I complex loaded with the TAA peptides migrate to the cell surface for presentation to the CTLs. **(B)** Maintaining protein homeostasis: Immunoproteasomes maintain protein homeostasis and protect cells from proteotoxic stress by degrading non-functional and misfolded proteins. **(C)** Degrading tumor suppressor protein IkBa: Immunoproteasome-mediated degradation of IkBa leads to NF-kB activation and cytokine secretion, which in turn recruits neutrophils and initiates colitis-associated cancers (CAC). **(D)** Promoting T cell differentiation: Immunoproteasomes trigger the differentiation of the pro-inflammatory Th1 and Th17 cells, resulting in increased production of IL-17, IL-22, TNF, and IFN-γ.

Enhancing immunoproteasome activity through cytokines, small molecules, or genetic modifications (**Figure 2**) can boost the ability of the immune system to recognize and destroy cancer cells (61). For instance, administration of IFN-γ and TNF-α, which are potent inducers of immunoproteasome subunits, have been shown to increase the expression and activity of the immunoproteasome (62). Furthermore, small molecules that mimic the effects of these cytokines or directly activate signaling pathways involved in immunoproteasome regulation are also under investigation. Finally, genetic engineering methods like CRISPR-Cas9 have been used to increase the expression of immunoproteasome subunits in tumor or immune cells, and enhance antigen processing and presentation (63–65).

### 4.2 Combination therapies

The combination of immunoproteasome inhibitors with immune checkpoint blockers represents a synergistic approach to cancer treatment. Antibodies targeting checkpoint molecules like PD-1, PD-L1, and CTLA-4 have been shown to enhance T cell-mediated elimination of cancer cells. However, the therapeutic efficacy of checkpoint inhibitors can be limited by the immunosuppressive TME and insufficient antigen presentation. Selective inhibition of the immunoproteasome can relieve the immunosuppression in some solid tumors by enhancing antigen presentation. Preclinical studies using animal models have shown that combining immunoproteasome inhibitors and checkpoint blockers prolonged the survival of tumor-bearing animals through enhanced anti-tumor activity. Furthermore, clinical trials are currently exploring this combination strategy in various cancers to evaluate its safety and therapeutic potential.

Immunoproteasome inhibitors can also augment the efficacy of adoptive cell therapies, such as the CAR-T cell therapy or TCR-engineered T cell therapy, by increasing the availability of target antigens within the TME. Similarly, cancer vaccines, which aim to elicit a strong immune response against tumor-specific antigens, can benefit from immunoproteasome inhibitors due to more effective presentation of vaccine-derived peptides. The activation and proliferation of effector T cells can also be increased by combining immunoproteasome inhibitors and cytokines like IL-2 or IL-15, resulting in a potent anti-tumor response.

### 4.3 Case studies of specific solid tumors

Bladder cancer is difficult to treat due to its recurrence and resistance to conventional therapies. Cathro et al. showed that immunoproteasome inhibitors synergistically improved the therapeutic effects of checkpoint blockade in an animal model of bladder carcinoma by increasing antigen presentation and infiltration of CTLs (66). Furthermore, several clinical trials are investigating the impact of combining immunoproteasome inhibitors with standard treatments, such as Bacillus Calmette-Guerin (BCG) therapy, on the overall immune response and recurrence rates in bladder cancer patients (67–69).

Bone cancers, including osteosarcoma and Ewing sarcoma, are aggressive malignancies that primarily affect children and young adults, and are difficult to treat due to their ability to evade the immune system. Niewerth et al. showed that immunoproteasome inhibitors sensitized bone cancer cells to immune-mediated destruction in a preclinical model by increasing the repertoire of presented antigens and promoting CTL infiltration. In addition to the direct destruction of tumor cells, this strategy also induced a pro-inflammatory TME with sustained immune surveillance. Current research efforts are focused on combining immunoproteasome-targeted therapies with other immunotherapies, such as CAR-T cell therapy, to achieve more favorable outcomes for bone cancer patients (70).

The immunoproteasome is also a promising therapeutic target for colorectal cancer (CRC). Studies have shown that upregulating immunoproteasome activity in CRC cells can improve antigen presentation and enhance the efficacy of immunotherapies, such as checkpoint blockade (11). Additionally, immunoproteasome inhibitors have been shown to reduce the immunosuppressive environment of CRC tumors by modulating cytokine production and inhibiting the function of regulatory T cells (Tregs) and myeloid-derived suppressor cells (MDSCs) (64, 71). At present, clinical trials are being conducted to assess the safety and efficacy of combining immunoproteasome-targeted therapies with chemotherapy and immunotherapy (72).

Due to its aggressive nature and the absence of targeted therapies, breast cancer, particularly triple-negative breast cancer (TNBC), presents significant treatment challenges. The immunoproteasome is a promising therapeutic target in breast cancer (73, 74). Studies show that immunoproteasome activity is crucial for the effective presentation of breast cancer-associated antigens and the activation of CTLs (75). Furthermore, immunoproteasome inhibitors have been shown to enhance antigen presentation and promote immune cell infiltration in preclinical models of breast cancer. These findings suggest that targeting the immunoproteasome could be an effective strategy to improve the efficacy of existing immunotherapies, such as checkpoint inhibitors, in breast cancer patients. Furthermore, in order to enhance the overall anti-tumor response and raise survival rates for patients with breast cancer, clinical trials are investigating the combination of immunoproteasome-targeted therapies with traditional treatments like radiation therapy and chemotherapy.

**Figure 3:**
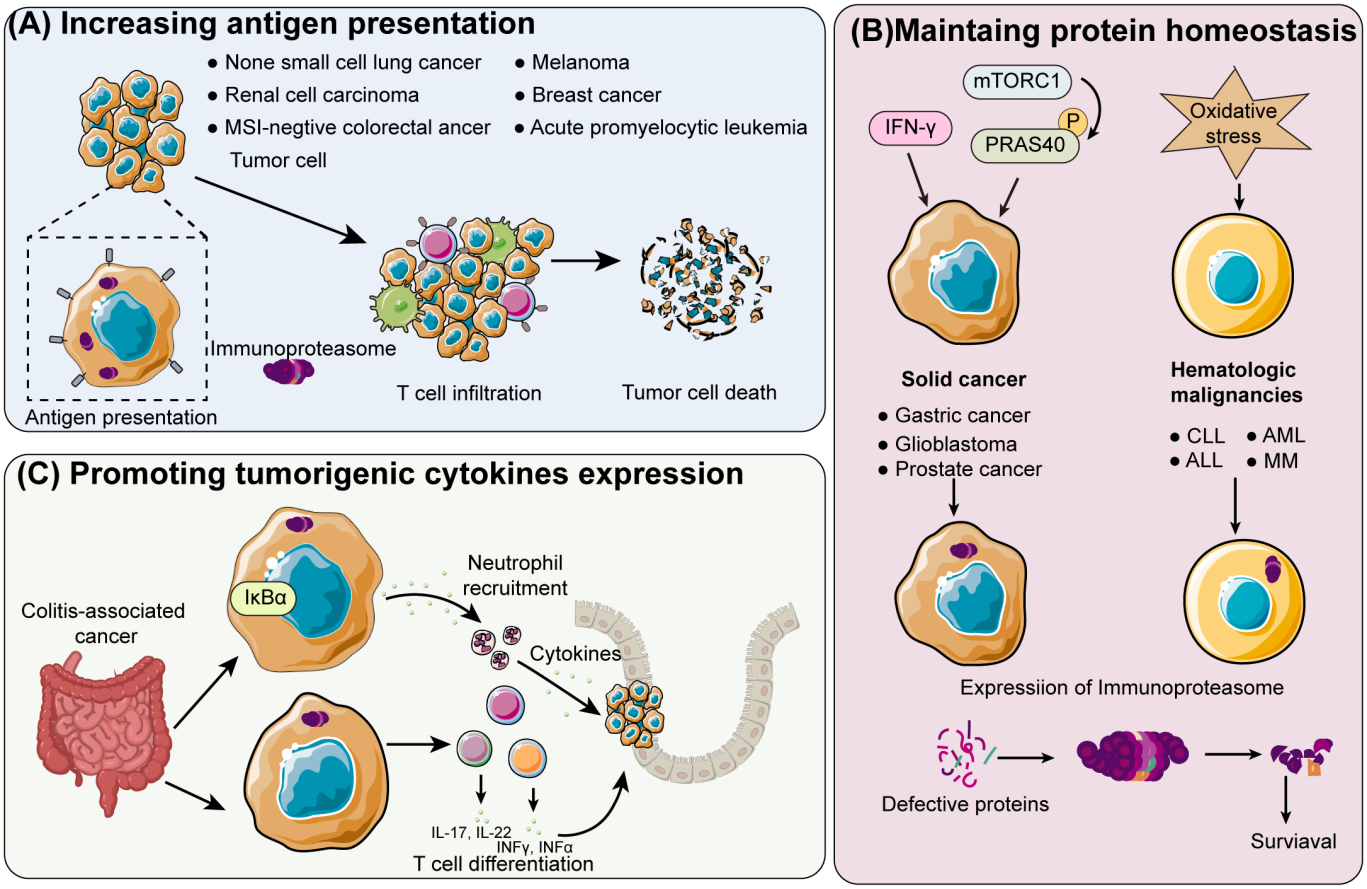
Schematic overview of immunoproteasome mechanisms in different cancer types **(A)** Enhancing tumor antigen presentation: The immunoproteasome enhances the presentation of tumor antigens in cancers like acute promyelocytic leukemia, breast cancer, non-small cell lung cancer, renal cell carcinoma, melanoma, and colorectal cancer that is negative for microsatellite instability (MSI). This leads to greater T-cell infiltration and subsequent tumor cell death. **(B)** Shifting proteasome population in solid and hematologic cancers: In solid cancers such as prostate, glioblastoma, and gastric cancer, the proteasome population is shifted towards immunoproteasomes by IFN-γ and phosphorylated proline-rich Akt substrate of 40 kDa (PRAS40), which is induced by hyperactivated mTOR complex 1 (mTORC1). Increased oxidative stress boosts immunoproteasome expression in hematologic malignancies, including multiple myeloma (MM), acute lymphoblastic leukemia (ALL), acute myeloid leukemia (AML), and chronic lymphocytic leukemia (CLL). Increased oxidative stress boosts immunoproteasome expression in hematologic malignancies, including multiple myeloma (MM), acute lymphoblastic leukemia (ALL), acute myeloid leukemia (AML), and chronic lymphocytic leukemia (CLL). Increased cancer cell survival is a result of the overexpressed immunoproteasome maintaining protein homeostasis. **(C)** Promoting tumorigenesis in colitis-associated cancer: The immunoproteasome degrades IκB in colitis-associated cancer, promoting the differentiation of T cells into the pro-tumorigenic inflammatory T helper cells.

Hepatocellular carcinoma (HCC) is one of the main causes of cancer-related death worldwide. Studies in preclinical models have shown that immuneproteasome inhibitors can increase the presentation of antigenic peptides and foster a pro-inflammatory TME, which can improve the effectiveness of immune checkpoint inhibitors, leading to a more effective activation of CTLs against liver cancer cells (76, 77). This combination approach has reduced tumor growth and improved survival rates in liver cancer models. Furthermore, immunoproteasome-targeted therapies in combination with other treatments, such as transarterial chemoembolization (TACE) and radiofrequency ablation (RFA), are also being investigated to enhance the overall anti-tumor response and improve clinical outcomes in liver cancer patients (**Figure 3**).

## 5 Clinical applications

Numerous clinical trials are currently investigating the efficacy, safety, and optimal use of immunoproteasome-targeted therapies, both inhibitors and enhancers, against various types of solid tumors. For example, several early-phase trials are evaluating the efficacy of immunoproteasome inhibitors such as ONX 0914 in combination with checkpoint inhibitors like pembrolizumab and nivolumab (78–80). In addition, preliminary results of some clinical trials show that a combination treatment of immunoproteasome inhibitors and anti-PD-1 antibody achieved better response rates and prolonged progression-free survival in patients with melanoma and lung cancer compared to monotherapy. Similarly, cytokine therapies that upregulate immunoproteasome activity have been shown to enhance the anti-tumor immune response. Therefore, targeting the immunoproteasome can improve the effectiveness of current immunotherapies and offer patients with solid tumors new alternatives. However, further studies are required to validate these results and determine the long-term benefits and safety profiles of these combination therapies.

The identification of reliable biomarkers that can predict patient response is essential for the clinical success of immunoproteasome-targeted therapies. For instance, the expression levels of immunoproteasome subunits (β1i, β2i, and β5i), cytokine profiles, and the presence of particular tumor antigens can predict therapeutic efficacy, as well as help screen patients who will most likely to benefit from these therapies. Furthermore, the immunoproteasome activity in tumor tissues and immune cells can serve as an indicator to guide the selection and optimization of immunoproteasome-targeted personalized therapies as per the unique tumor and immune system characteristics of individual patients. For instance, patients with low baseline immunoproteasome activity may benefit from therapies that upregulate its function. In contrast, those with high activity might respond better to inhibitors that modulate their activity. Thus, personalized treatment strategies can enhance the efficacy and minimize the side effects of immunoproteasome-targeted therapies, leading to more effective and individualized cancer care.

## 6 Challenges and future directions

Immunoproteasome inhibitors can also target the proteasomes in normal cells, resulting in the accumulation of damaged or misfolded proteins, which trigger cellular stress and apoptosis. In fact, several side effects of immunoproteasome inhibitors have been reported in clinical trials, such as fatigue, gastrointestinal issues, and hematological toxicities. Developing inhibitors with greater specificity for cancer cells or optimizing dosing regimens can minimize these adverse effects. Furthermore, cancer cells can develop resistance to immunoproteasome inhibitors through various mechanisms, such as upregulation of compensatory proteolytic pathways, mutation of target subunits, or altered expression of proteasome-related genes, leading to relapse and treatment failure. Therefore, it is crucial to elucidate these mechanisms and develop novel strategies to overcome resistance to immunoproteasome inhibitors, such as combining other therapeutic agents that target complementary pathways or using sequential treatment approaches.

Future research should also focus on identifying novel targets within the immunoproteasome pathway. High-throughput screening and advanced genomic techniques can facilitate the discovery of new subunits or regulatory proteins. Additionally, understanding the complex interactions between the immunoproteasome and other cellular pathways can reveal new therapeutic opportunities. For instance, targeting regulatory proteins that control immunoproteasome assembly and activity could provide alternative strategies for modulating its function. Furthermore, advances in drug delivery systems can significantly enhance the efficacy and safety of immunoproteasome-targeted therapies. Nanotechnology-based delivery systems, such as liposomes, nanoparticles, and micelles, can improve the selective targeting of immunoproteasome inhibitors to tumor cells while sparing normal tissues. These systems can also provide controlled and sustained release of the therapeutic agents, reducing the frequency of administration and improving patient compliance. Finally, developing targeted delivery systems that exploit tumor-specific markers or the unique microenvironment of tumors can enhance the precision and effectiveness of immunoproteasome inhibitors.

Immunoproteasome-targeted therapies also hold promise for hematological malignancies such as multiple myeloma and lymphoma due to the high immunoproteasome activity that is frequently observed in these cancers. Exploring the efficacy of these therapies in a broader range of cancers can uncover new clinical applications and benefit a larger patient population. Furthermore, the development of more tailored and effective treatments requires a greater understanding of the differential expression and activity of immunoproteasomes across various cancer types. There is also evidence of a regulatory role of the immunoproteasome in autoimmune diseases and persistent infections. Thus, blocking immunoproteasome activity in autoimmune diseases could mitigate the aberrant immune responses, and reduce inflammation and tissue damage. For instance, immunoproteasome inhibitors have shown promising results in preclinical models of rheumatoid arthritis and lupus. Similarly, enhancing immunoproteasome activity could boost the immune response against chronic infections by improving antigen presentation and T-cell activation. Future research should explore these novel therapeutic possibilities for autoimmune diseases and chronic infections.

In conclusion, the clinical translation of immunoproteasome-targeted therapies will rely on addressing toxicity and resistance mechanisms and exploring novel targets and delivery systems, in order to optimize the therapeutic potential of the immunoproteasome.

## 7 Conclusion

The immunoproteasome is a key determinant of the response to cancer immunotherapy as it ensures the activation of anti-tumor CTLs by promoting generation of antigenic peptides and their presentation on MHC class I molecules, which in turn improves identification and elimination of cancer cells. Goven its role in modulating the TME and anti-tumor immune responses, the immunoproteasome has garnered considerable interest as a therapeutic target for cancer treatment. Recent studies have greatly improved our understanding of its structure, function, and regulation. Furthermore, clinical trials have reported promising results of immunoproteasome inhibitors, such as ONX 0914, against melanoma and lung cancer. Immunoproteasome-targeted therapies have also been shown to enhance the efficacy of checkpoint blockade therapy and improve patient outcomes. Additionally, strategies that upregulate immunoproteasome activity are also being explored to boost immune responses against tumors.

Future studies should focus on comprehending the mechanisms underlying immunoproteasome inhibitor resistance and developing strategies to address these challenges. Additionally, identifying reliable biomarkers for predicting patient response to immunoproteasome-targeted therapies will be crucial for personalizing treatment plans and maximizing therapeutic efficacy. Novel drug delivery systems and integrating immunoproteasome-targeted therapies with existing treatment regimens could offer new hope for patients with solid tumors, particularly those who do not respond well to conventional therapies. Tailoring treatments based on immunoproteasome activity and patient-specific biomarkers can enhance the precision and effectiveness of cancer therapy, leading to better patient outcomes and reduced side effects.

In conclusion, immunoproteasome-targeted therapies can enhance immune responses and overcome tumor-induced immunosuppression, resulting in more effective and durable treatment for cancer patients.
